# Open source code for the generation of digital reference objects for dynamic contrast-enhanced MRI analysis software validation

**DOI:** 10.1259/bjr.20220976

**Published:** 2023-05-25

**Authors:** Andrew B. Gill, Ferdia A. Gallagher, Martin J. Graves

**Affiliations:** 1 Department of Radiology, University of Cambridge, Cambridge, UK

## Abstract

**Objectives:**

Dynamic contrast-enhanced MR images can be analyzed through the application of a wide range of kinetic models. This process is prone to variability and a lack of standardization that can affect the measured metrics. There is a need for customized digital reference objects (DROs) for the validation of DCE-MRI software packages that undertake kinetic model analysis. DROs are currently available only for a small subset of the kinetic models commonly applied to DCE-MRI data. This work aimed to address this gap.

**Methods:**

Code was written in the MATLAB programming environment to generate customizable DROs. This modular code allows the insertion of a plug-in to describe the kinetic model to be tested. We input our generated DROs into three commercial and open-source analysis packages and assessed the agreement of kinetic model parameter values output with the ‘ground-truth’ values used in the DRO generation.

**Results:**

For the five kinetic models tested, the concordance correlation coefficient values were >98%, indicating excellent agreement of the results with ‘ground-truth’.

**Conclusions:**

Testing our DROs on three independent software packages produced concordant results, strongly suggesting our DRO generation code is correct. This implies that our DROs can be used to validate other third party software for the kinetic model analysis of DCE-MRI data.

**Advances in knowledge:**

This work extends published work of others to allow customized generation of test objects for any applied kinetic model and allows the incorporation of B_1_ mapping into the DRO for application at higher field strengths.

## Introduction

It has previously been documented^1–3^ that the complex analysis of dynamic contrast-enhanced MR imaging (DCE-MRI) data using kinetic modeling techniques can involve significant variability, and lack of standardization. This has restricted the translation of DCE-MRI from a research technique into successful application in the clinic.^4^ Recently, the Quantitative Imaging Biomarkers Alliance within the RSNA (QIBA) has been established to explore this lack of standardization and other variability, and to establish common protocols and procedures to eliminate it.^5^

One source of variability in DCE-MRI kinetic model analysis is the lack of standardized analysis software. There are many software packages available for such analyses, both commercial and open source, and many institutions develop their own bespoke solutions. One author has concluded that software variability, whether due to errors in implementation or variability in design, can be responsible for differences in output kinetic model parameters of up to 74% (within subject coefficient of variation) when presented with identical input data.^6^

The availability of suitable input reference data is paramount to the testing and validation of such software packages. In 2009, QIBA made a set of digital reference objects (DROs) available which simulate *in silico* DCE-MRI input data with known kinetic model parameter output.^7^ Each QIBA DRO is delivered as a set of DICOM images that can be used as input into almost any DCE-MRI analysis package.

Although the QIBA DROs are useful, they are only currently available for the Tofts and extended Tofts kinetic models, which limits their wider application. The fact that they have been generated using a third party simulation software package, JSim,^8^ which may not be familiar to many readers, makes it difficult to customize or amend these DROs. In addition, they do not account for the full complexity of DCE-MRI data acquired in some experimental conditions: for example, the incorporation into the acquisition protocol of a sequence that can generate a B_1_ map, is now considered essential when performing DCE-MRI at high field strengths (typically ≥ 3 T). In addition, the customization of the arterial input function (AIF) used in the generation of the QIBA DROs is not easy to undertake.

In summary, there is a current need for publicly available DROs, similar in format to the QIBA DROs, that take account of the whole range of kinetic analysis models commonly in use, including reference B_1_ maps as well as baseline T_1_ (T_1,0_) maps (or the equivalent source images from *e.g*. variable flip angle (VFA) acquisitions) along with the dynamic series itself.

This paper presents open-source MATLAB (Mathworks, Natick, MA) code that is designed to generate configurable DICOM DROs with these attributes. The code is general in purpose and presented in a modular format in order to encourage modification and extension to suit the user’s particular requirements. It is primarily designed as a tool for quality control and validation of DCE-MRI analysis software packages, although it could also be used for simulation studies where one or more aspects of the resulting digital phantoms are varied for investigational purposes.

## Methods and materials

A generalizable and customizable set of scripts was developed in MATLAB for the generation of DCE-MRI digital reference objects.

The analysis of such DCE-MRI image data via a kinetic model typically proceeds via a number of stages. The first stage is the conversion of dynamic image intensities (gray-scale levels indicative of acquired MR signals) to concentration-time curves, representing the evolution of contrast agent concentration (typically gadolinium based) in each imaged voxel. In a typical DCE-MRI acquisition, this is achieved through correction of the images for imperfect excitation flip angles using B_1_ mapping techniques. There then follows computation of the T_1_ relaxation time (or equivalently R_1_ relaxation rate) evolution over time via the standard SPGR equation^9^ that relates the tissue T_1_ with the acquired signal intensity. A relaxivity relationship is then applied to yield the required contrast agent concentration time (CACT) curve.

Secondly, a suitable kinetic model is fitted to the CACTs yielded at each voxel and a reference curve, the arterial input function, which is the CACT measured in the artery supplying blood to the tissues in the imaged voxels.

Finally, application of the kinetic model to the CACTs, usually incorporating curve fitting via an optimization algorithm, yields physiologically meaningful parameter values for each imaged voxel.

The algorithm followed to generate DROs reverses the steps above, leading to the construction of synthetic DCE-MRI images from known kinetic model parameter values. This allows subsequent analysis of the synthetic images to compare the output with the known input, and hence to achieve validation of the analysis software used.

**Figure 1.**
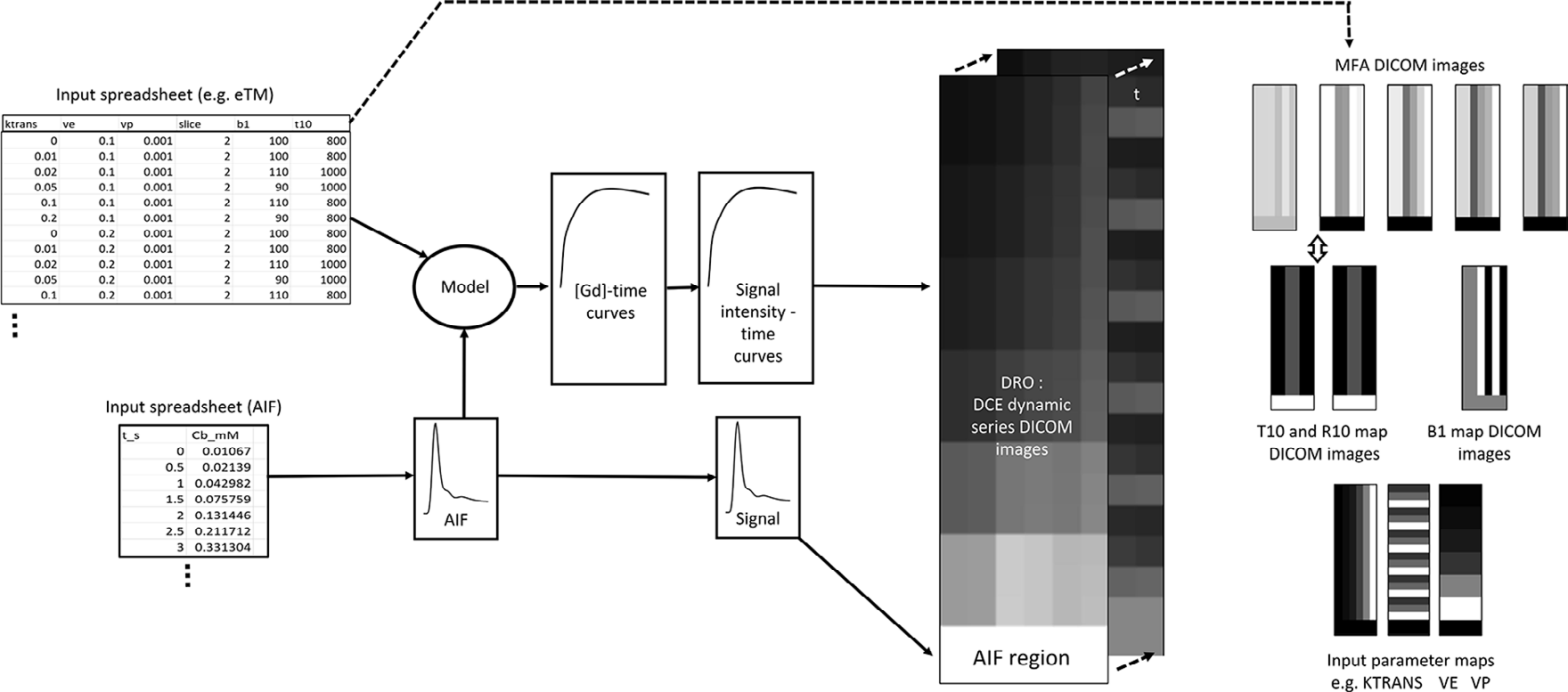
Input kinetic model parameter values are entered to our software via a ‘.csv’ format spreadsheet along with B_1_ and T_1,0_ map values; the extended Tofts model is chosen here by way of illustration. The AIF is also input from a similar spreadsheet. Using the AIF, input T_1,0_ and kinetic model parameters, [Gd]-time curves are calculated from the kinetic model in question. The [Gd]-time curves are converted to signal-time curves through application of the standard SPGR equation. Finally, a digital reference object is output consisting of a 4-D DICOM image set representing DCE-MRI images evolving in the time domain. Also output in DICOM format are input parameter maps, T_1,0_ (and R_1,0_) maps, along with multiple flip angle images that can be used to generate these maps and B_1_ maps.

MATLAB code was developed to generate customizable DCE-MRI DROs. This code was designed with the following characteristics (Figure 1):

An input domain of kinetic model index values is specified along with parameters defining the layout and composition of the resulting DRO. Also configured are the various parameters associated with the MR acquisition being simulated (*e.g.,* TR, TE, time resolution, duration, etc.) and the synthetic ‘tissues’ under study (native T_1_ values, baseline signal values, relaxivity constants, etc.). In addition, the AIF to be used in the DRO generation is specified as a ‘blood-concentration’.

For each voxel in the DRO, the kinetic model under investigation is applied in the ‘forwards direction’ using the associated known kinetic model indices and the given AIF. The kinetic model is specified via a modular plug-in script containing the residue function specific to the applied model expressed in convolutional form. A contrast-agent concentration-time curve is output from this process, one for each voxel in the tissue to be simulated.

In turn, the CACTs are then converted into MR signal grey-level curves (signal-time curves) by reverse application of the standard relaxivity and spoiled gradient echo (SPGR) equations as implemented by Schabel & Parker.^10^ Finally, a 4D dynamic DICOM image set is exported with grey-level evolution in time at each voxel as specified by the signal-time curve.

A T_1_ (and the equivalent R_1_) map is generated for the synthetic tissue volume along with a set of variable flip-angle images which can be used for generation of this T_1_ map. Finally, the associated B_1_ map, used in calculating the voxel flip-angles for the dynamic series and T_1,0_ maps, is also output in DICOM format.

For completeness, the input known value kinetic parameter maps are also output in DICOM format. Suitable DICOM headers are attached to all output images, ensuring that they may be read by most DCE-MRI analysis software packages taking input in DICOM format.

The resulting DRO output volume can be configured to have any size and one or more representative MR image slices.

Commonly used kinetic models, especially in tumor studies, include the Tofts model^11,12^ and the extended Tofts model^13^ which yield the parameters *K* ^trans^, *v*_e_ and, in the latter case, *v*_p_. Other models, for example the Patlak model,^14^ a tissue uptake model,^15^ and a two-compartment exchange model (2CXM),^16^ have also been employed where there is an *a priori* theoretical reason to expect their better representation of the physiology and applied acquisition regime (see^17–19^ for further details).

Example DROs were generated using our MATLAB code for the Tofts model, the extended Tofts model, the Patlak model, a tissue uptake model and a two-compartment exchange model. The residue function for each was taken from the work of Sourbron & Buckley^17^ as implemented in MATLAB in Barnes *et al*.^20^ The arterial input function used was as described in the QIBA DRO documentation^7^ and is provided as a ‘.csv’ file with the source code.

Application of the model using a residue function requires numerical integration. In our examples, this integration was implemented using a trapezoidal algorithm. To facilitate accurate numerical calculation for DRO’s subsequently down-sampled to a low temporal resolution, we allow the user to specify a time resolution for the integration process that may be different from that of the resulting dynamic series. In our examples, both the integration interval and the dynamic series time resolution were set to 0.5 s.

The configuration parameters of each DRO are summarized in Table 1 and the domain of kinetic index parameter values, in each case typical of those reported in tumor studies, is supplied as ‘.csv’ files with the source code.

**Table 1.** Configuration parameters for sample digital reference objects.

DRO parameter	Value
Time resolution (s)	0.5
Dynamic series timepoints	650
Dynamic series flip angle (degrees)	25
Estimated hematocrit	0.45
Baseline points	10
Tissue delay (s)	0
Gd dose (mmol kg^−1^)	0.1
Plasma relaxivity (mM^−1^ s^−1^)	4.5
TR (ms)	5
Pixel dimensions (mm)	1
Slice thickness (mm)	10
Pixels per block	10
Blood T_1,0_ (ms)	1664

The example DRO DICOM datasets were then used as input into three DCE-MRI analysis software packages: the ROCKETSHIP open-source software developed in MATLAB by Barnes *et al* ^20^; and the commercially available MIStar package (version 3.2.63) (Apollo Medical Imaging Technology, Melbourne, Australia); and the MADYM software (version 4.16.1) developed in *C* ++ by Berks *et al*.^21^ Starting values for the kinetic model optimization process are given in Table 2.

**Table 2.** Initial values for kinetic model parameters input into analysis code

	*K* ^trans^ [min^−1^]	*v_e_*	*v*_p_	F_p_ [ml min^−1^ ml^−1^]	PS [min^−1^]
**Patlak**	0.1	0.25	0.02	n/a	n/a
**TM**	0.1	0.25	n/a	n/a	n/a
**eTM**	0.1	0.25	0.02	n/a	n/a
**TU**	n/a	n/a	0.05	0.5	0.125
**2CXM**	n/a	0.25	0.05	0.5	0.125

In the case of ROCKETSHIP and MADYM, there is a requirement to input images in NIfTI format. This was achieved using a third party DICOM-to-NIfTI conversion tool (dcm2niiX.exe)^22^ in the case of ROCKETSHIP, and using the supplied conversion tool ‘madym_DicomConvert’ in the case of MADYM. Sample conversion scripts for the latter case are supplied with the source code.

Agreement between output kinetic model parameter values and the known true values was displayed in the form of concordance plots, and expressed numerically in terms of the concordance correlation coefficient (CCC)^23^ with 95% confidence intervals.

Examples of non-perfect concordance were investigated by running the analysis software packages with differing starting values for each of the modeled parameters.

Additional DROs were also generated and tested for the eTM and 2CXM models using different dynamic series time resolutions and integration intervals, to investigate any resolution dependent effects.

## Results

The essential features of one example DRO generated by our code are shown in Figure 1.

The open source MATLAB code itself is available at https://github.com/anstepsa/dce-dro-matlab.

**Figure 2.**
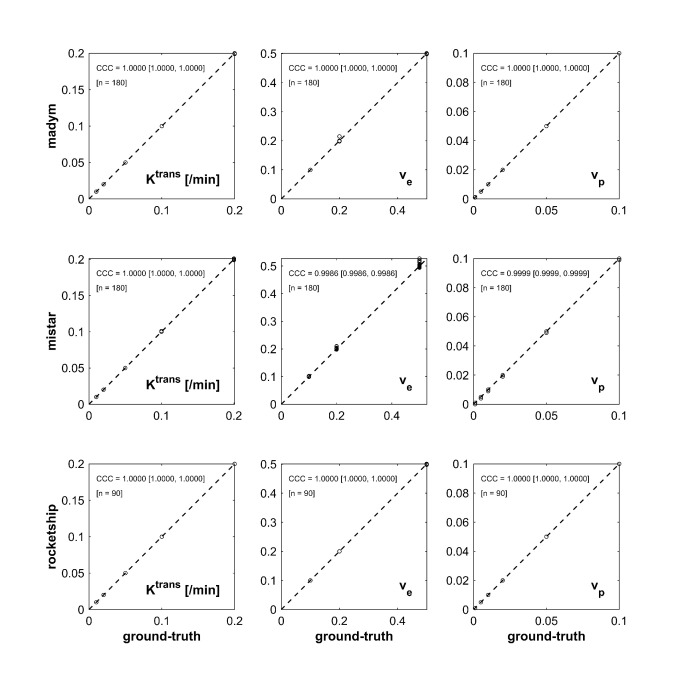
Concordance plots expressing agreement between output parameter values from generated ‘extended Tofts model’ DRO input into three software packages: (upper row) MADYM, (center row) MIStar, (lower row) ROCKETSHIP.

**Figure 3.**
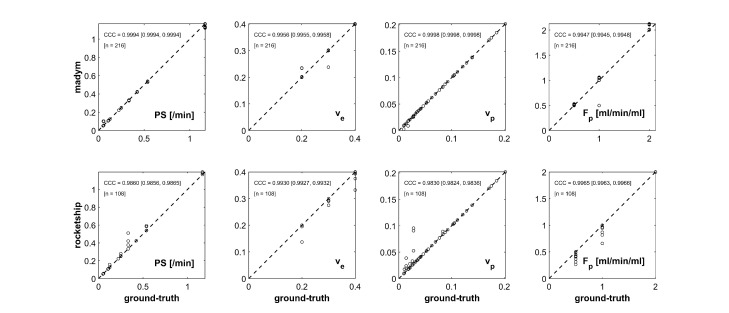
Concordance plots expressing agreement between output parameter values from generated ‘two-compartment exchange model’ DRO input into two software packages: (upper row) MADYM, (lower row) ROCKETSHIP.

Sample results are shown in Figures 2 and 3 as concordance plots (drawn using a custom MATLAB script) and expressing the agreement between the kinetic parameter maps output by each analysis platform, and true parameter values (*i.e.,* those used to generate the DROs in the first place).

Table 3 shows CCC values for all parameters for all models in the three analysis packages tested. The CCC values are >99% in all but one case which indicates almost perfect agreement^24^ with the known true values in every model and software package. Note that for the eTM, voxels with a true *K* ^trans^ of 0.0 are excluded from the plotted data in Figure 2. Convergence of the model was typically poorer for this subset of voxels representing a largely non-physical case.

**Table 3.** Lin’s concordance correlation coefficient (CCC) values for comparison of kinetic model parameter output from DROs generated for five example kinetic models for three software packages tested. *n/a* = not applicable (model not supported for analysis in the software package)

Model	Parameter	MIStar (CCC)		ROCKETSHIP (CCC)		MADYM (CCC)	
Patlak	*K* ^trans^	1.0000	[1.0000, 1.0000]	1.0000	[1.0000, 1.0000]	n/a	
Patlak	*v*_p_	0.9995	[0.9995, 0.9995	1.0000	[1.0000, 1.0000]	n/a	
Tofts	*K* ^trans^	0.9998	[0.9998, 0.9998]	1.0000	[1.0000, 1.0000]	1.0000	[1.0000, 1.0000]
Tofts	*v_e_*	1.0000	[1.0000, 1.0000]	1.0000	[1.0000, 1.0000]	1.0000	[1.0000, 1.0000]
ext. Tofts	*K* ^trans^	1.0000	[1.0000, 1.0000]	1.0000	[1.0000, 1.0000]	1.0000	[1.0000, 1.0000]
ext. Tofts	*v_e_*	0.9986	[0.9986, 0.9886]	1.0000	[1.0000, 1.0000]	1.0000	[1.0000, 1.0000]
ext. Tofts	*v*_p_	0.9999	[0.9999, 0.9999]	1.0000	[1.0000, 1.0000]	1.0000	[1.0000, 1.0000]
TU	*PS*	n/a		0.9962	[0.9960, 0.9964]	n/a	
TU	*v*_p_	n/a		0.9923	[0.9917, 0.9928]	n/a	
TU	F_p_	n/a		0.9996	[0.9996, 0.9996]	n/a	
2CXM	*PS*	n/a		0.9860	[0.9856, 0.9865]	0.9994	[0.9994, 0.9994]
2CXM	*v_e_*	n/a		0.9930	[0.9927, 0.9932]	0.9956	[0.9955, 0.9958]
2CXM	*v*_p_	n/a		0.9830	[0.9824, 0.9836]	0.9998	[0.9998, 0.9998]
2CXM	F_p_	n/a		0.9965	[0.9963, 0.9966]	0.9947	[0.9945, 0.9948]

We can conclude from this that, in all likelihood, each software package is correct and that the DROs generated by our code are correct.

The CACT curve and the fitted curve were plotted for each of the data-points not lying on the concordance line in Figures 2 and 3. It was found that perfect, or in a minority of cases near-perfect, concordance could be restored by changing the initial values of the fitted parameters used in the optimization process. This would indicate that local minima found in optimization were the primary cause of non-perfect concordance of parameter values. Further detail on this can be found in the Supplementary Material 1.

Supplementary Material 1.

Table 4 shows the CCC values for DROs generated with varying time resolution and analyzed for constant initial parameter estimates and varying time intervals used in integration. For dynamic series time resolutions (dt) of up to 5 s, the eTM was analyzed with excellent concordance by all three software packages. At a dynamic series time resolution of 7.5 s, MADYM’s automatic AIF recognition algorithm failed; ROCKETSHIP showed slight degradation in CCC values although still excellent concordance. The 2CXM showed greater variability: MADYM displayed slightly degraded CCC values as dt was increased to 5 s, when integrals were performed using a time resolution (integral dt) of 0.5 s, and greatly diminished CCC for dt > 2 s when dt and integral dt were matched. ROCKETSHIP performed with excellent CCC when dt and integral dt were matched, no doubt because the ROCKETSHIP analysis algorithm then exactly matched that of the DRO generation process.

**Table 4.** Lin’s concordance correlation coefficient (CCC) values for comparative analyses of kinetic model parameter output from DROs generated with differing time-resolution (‘dt’) and differing integration intervals (‘integration dt’)

CCC	dt (s)		0.5	2	5	7.5	0.5	2	5
	integration dt (s)		0.5	0.5	0.5	0.5	0.5	2	5
ext. Tofts	MADYM	*K* ^trans^	1.0000	0.9999	0.9990	no AIF found^a^	1.0000	1.0000	0.9998
		v_e_	1.0000	0.9999	0.9991		1.0000	1.0000	0.9998
		v_p_	1.0000	0.9999	0.9979		1.0000	0.9999	0.9994
	ROCKETSHIP	*K* ^trans^	1.0000	1.0000	0.9999	0.9993	1.0000	1.0000	1.0000
		v_e_	1.0000	1.0000	0.9993	0.9981	1.0000	0.9945	0.9959
		v_p_	1.0000	1.0000	1.0000	0.9998	1.0000	1.0000	1.0000
	MIStar	*K* ^trans^	1.0000	1.0000	0.9960	0.9969	1.0000	1.0000	0.9878
		v_e_	0.9986	0.9998	0.9903	0.9973	0.9986	0.9995	0.9929
		v_p_	0.9999	0.9999	0.9974	0.9871	0.9999	0.9998	0.9960
2CXM	MADYM	*PS*	0.9994	0.9931	0.9647	not measured	0.9994	0.9595	0.8465
		v_e_	0.9956	0.9936	0.9428		0.9956	0.9943	0.9691
		v_p_	0.9998	0.9948	0.9154		0.9998	0.9950	0.8945
		F_p_	0.9947	0.9950	0.9819		0.9947	0.5535	0.0549
	ROCKETSHIP	*PS*	0.9841	0.9784	0.8535		0.9841	0.9973	0.9998
		v_e_	0.9858	0.9900	0.8771		0.9858	0.9990	1.0000
		v_p_	0.9897	0.9773	0.7614		0.9897	0.9976	0.9999
		F_p_	0.9942	0.9076	0.6550		0.9942	0.9999	1.0000

a No arterial input function could be determined by MADYM for this dataset.

In Figures 2 and 3 the number (‘n’) of distinct parameter combinations analyzed is fewer in ROCKETSHIP than in MADYM or MIStar since the former cannot include a B_1_ map in the analysis process.

## Discussion

Open source MATLAB code has been presented here for the generation of customizable and generalized digital reference objects for the kinetic model analysis of DCE-MRI data. This code is offered for the production of validation *in silico* phantoms, although this use could be extended to more detailed simulation studies. Five sample DROs were generated and shown to be correct in the sense that they yielded results in excellent agreement with the known input values when analyzed by three separate third-party analysis software packages.

DCE-MRI DROs have been presented in the literature before, most importantly with the publication of the QIBA DROs^7^ to be used as a reference standard. The purpose of this work is not to replace the QIBA DROs, but to complement and extend them with a means of DRO generation and customization open to any reader who is familiar with the MATLAB development environment.

Several recent publications have reported on various attempts to generate DROs for quality control and investigational purposes in DCE-MRI. Beers *et al* ^25^ report anatomically realistic DROs generated from clinical MR image datasets of glioma patients using only the Tofts model. The resulting ‘noise-free’ dynamic images are then analyzed with varying degrees of random noise added for quality control purposes. Bernal *et al* ^26^ follow a similar approach to the production of DROs for the Patlak model to investigate blood-brain barrier leakage, incorporating more sophisticated noise addition methods (in the k-space domain) and known motion artefact methods. Semmineh *et al* ^27^ take a different approach by using a tissue structure model and real brain perfusion imaging data to generate DROs for quality control and investigational purposes in dynamic susceptibility contrast (DSC) MRI. Bleisener *et al* ^28^ investigate the effect of k-t sampling on DCE-MRI kinetic parameter results using the Patlak and extended Tofts models with simulated brain tumor DROs. These DROs were generated following the methodology of Bosca *et al*,^29^ which uses the extended Tofts model and segmented representative clinical brain MR images. Debus *et al* ^30^ make available a DRO generated using JSim for the 2CXM and use the QIBA DROs for the Tofts and extended Tofts models in the validation of their MITK platform for DCE-MRI analyses. Hansen *et al* ^31^ generate DROs for the Tofts, extended Tofts, and 2CXM models to validate a Bayesian DCE-MRI analysis method. Our current work presents a unifying and generalizable code base to generate DROs similar in type to those mentioned in the above studies.

There are limitations to our DRO study and code base. We are not aware of any true gold standard in DCE-MRI kinetic model analysis. Although physical perfusion phantoms have been developed^32,33^ to validate CACT curves calculated from DCE-MRI experiments, these do not directly validate any known kinetic parameter associated with the phantom (*e.g., K* ^trans^).

The use of kinetic model code in generating a DRO inevitably leads to the possibility of variability in the DRO images according to the algorithms employed. Since the coding of the kinetic model algorithms in our DRO code follows the ROCKETSHIP implementation, they might be expected to yield good validation results when analyzed using the ROCKETSHIP software. This is less expected in the case of the MIStar and MADYM software packages where details of the kinetic model implementation are either unpublished (MIStar) or independently implemented (in *C* ++ in MADYM) and hence not studied by the authors of this paper.

It was found that the degree of agreement of results with the known true values was to some extent dependent on the starting values of the kinetic model parameters in the associated optimization process. This is as expected in general in optimization processes and was especially apparent for the 2CXM case with four free parameters.

With the proviso that the DROs were generated using a dynamic series (and AIF) time resolution of less than 5 s, they were found to yield excellent concordance with known values when analyzed by three independent software packages. The dependence of analysis results on the time-resolution of the dynamic series (and especially of the AIF) is to be expected and was more pronounced in the 2CXM than the eTM, no doubt again due to the inherent complexities of fitting a greater number of free parameters. Interestingly, the ROCKETSHIP analysis showed no degradation of CCC values with dynamic series time resolution when the integrals were calculated with a matched time interval. We can hypothesize that this is because the ROCKETSHIP algorithm was most nearly matched to the DRO generation algorithm in these circumstances (*i.e.,* trapezoidal integration at the time resolution of the dynamic series). MADYM showed pronounced failure of CCC in the same (poor) DRO generation conditions indicating that its analysis algorithm may more correctly interpolate the CACT curves before integration. When the DRO was generated more correctly using a small integration time interval, MADYM performed with much greater concordance. The user of our software should therefore select time resolution of generated DROs with care: a dynamic series/AIF time resolution of ≤2 s and an integral time resolution of ≤0.5 s is recommended for the most consistent results.

It can be noted that the synthetic DCE-MRI images produced by our code are free from noise or artefacts and can be relied upon to yield kinetic parameter indices without complications arising from typical imperfections present in actual *in vivo* imaging. This is both an advantage, in that any error in analysis is therefore not obscured or confounded by errors in acquisition, and a disadvantage in that the scope for realistic simulation studies is diminished. A rudimentary capability for adding noise to the generated DRO images is provided in our software although realistic simulation is not the primary aim of this study. In generating the DROs we have applied the kinetic model to the CACT curves rather than the signal intensity time curves themselves. This could introduce unwelcome non-linearities in error propagation in signals that include noise. Therefore, use of these DROs for simulation studies would not especially be recommended; comprehensive simulation studies would be coded much more efficiently through other means.

The user may need to edit or augment the supplied DICOM header information for any particular analysis application. This can be accomplished easily in the MATLAB programming environment or indeed using many commonly available DICOM viewers.

Conversion of our DROs to NIfTI format^34^ is also easily accomplished using third party DICOM-to-NIfTI converters. The reverse conversion (NIfTI-to-DICOM) is seldom so straightforward, which is the reason we chose to export our DROs as DICOM objects.

## Conclusions

We have presented here a substantially validated tool for quality control and evaluation of DCE-MRI kinetic model analysis packages. Our code to generate DROs is in a widely known format (MATLAB), and is easily generalizable and customizable. We also make available in DICOM format some example DROs constructed for common kinetic models in clinically realistic scenarios.

## References

[1] Kim H . Variability in quantitative DCE-MRI: sources and solutions. J Nat Sci 2018; 4(): e484. 29527572 PMC5841165

[2] Huang W, Li X, Chen Y, Li X, Chang M-C, Oborski MJ, et al . Variations of dynamic contrast-enhanced magnetic resonance imaging in evaluation of breast cancer therapy response: a multicenter data analysis challenge. Transl Oncol 2014; 7: 153–66. doi: 10.1593/tlo.13838 24772219 PMC3998693

[3] Kudo K, Christensen S, Sasaki M, Østergaard L, Shirato H, Ogasawara K, et al . Accuracy and reliability assessment of CT and MR perfusion analysis software using a digital phantom. Radiology 2013; 267: 201–11. doi: 10.1148/radiol.12112618 23220899 PMC3606546

[4] O’Connor JPB, Aboagye EO, Adams JE, Aerts HJWL, Barrington SF, Beer AJ, et al . Imaging biomarker roadmap for cancer studies. Nat Rev Clin Oncol 2017; 14: 169–86. doi: 10.1038/nrclinonc.2016.162 27725679 PMC5378302

[5] Radiological Society of North America . Quantitative Imaging Biomarkers Alliance (QIBA). Available from: https://www.rsna.org/QIBA/ (accessed 17 Sep 2021)

[6] Heye T, Davenport MS, Horvath JJ, Feuerlein S, Breault SR, Bashir MR, et al . Reproducibility of dynamic contrast-enhanced MR imaging. Part I. perfusion characteristics in the female pelvis by using multiple computer-aided diagnosis perfusion analysis solutions. Radiology 2013; 266: 801–11. doi: 10.1148/radiol.12120278 23220897

[7] QIBA digital reference objects: Barboriak Lab., Duke University. Available from: https://sites.duke.edu/dblab/qibacontent/ (accessed 17 Sep 2021)

[8] Butterworth E, Jardine BE, Raymond GM, Neal ML, Bassingthwaighte JB . JSim, an open-source modeling system for data analysis. F1000Res 2014; 2: 288. doi: 10.12688/f1000research.2-288.v3 PMC3901508 24555116

[9] Buxton RB, Edelman RR, Rosen BR, Wismer GL, Brady TJ . Contrast in rapid MR imaging: T1- and T2-weighted imaging. J Comput Assist Tomogr 1987; 11: 7–16. doi: 10.1097/00004728-198701000-00003 3805431

[10] Schabel MC, Parker DL . Uncertainty and bias in contrast concentration measurements using spoiled gradient echo pulse sequences. Phys Med Biol 2008; 53: 2345–73. doi: 10.1088/0031-9155/53/9/010 18421121 PMC2894639

[11] Tofts PS, Kermode AG . Measurement of the blood-brain barrier permeability and leakage space using dynamic MR imaging. 1. fundamental concepts. Magn Reson Med 1991; 17: 357–67. doi: 10.1002/mrm.1910170208 2062210

[12] Tofts PS . Modeling tracer kinetics in dynamic Gd-DTPA MR imaging. J Magn Reson Imaging 1997; 7: 91–101. doi: 10.1002/jmri.1880070113 9039598

[13] Tofts PS, Brix G, Buckley DL, Evelhoch JL, Henderson E, Knopp MV, et al . Estimating kinetic parameters from dynamic contrast-enhanced T (1) -weighted MRI of a diffusable tracer: standardized quantities and symbols. J Magn Reson Imaging 1999; 10: 223–32. doi: 10.1002/(sici)1522-2586(199909)10:3<223::aid-jmri2>3.0.co;2-s 10508281

[14] Patlak CS, Blasberg RG . Graphical evaluation of blood-to-brain transfer constants from multiple-time uptake data. generalizations. J Cereb Blood Flow Metab 1985; 5: 584–90. doi: 10.1038/jcbfm.1985.87 4055928

[15] de Bazelaire C, Siauve N, Fournier L, Frouin F, Robert P, Clement O, et al . Comprehensive model for simultaneous MRI determination of perfusion and permeability using a blood-pool agent in rats rhabdomyosarcoma. Eur Radiol 2005; 15: 2497–2505. doi: 10.1007/s00330-005-2873-z 16132928

[16] Sourbron S, Ingrisch M, Siefert A, Reiser M, Herrmann K . Quantification of cerebral blood flow, cerebral blood volume, and blood-brain-barrier leakage with DCE-MRI. Magn Reson Med 2009; 62: 205–17. doi: 10.1002/mrm.22005 19449435

[17] Sourbron SP, Buckley DL . Tracer kinetic modelling in MRI: estimating perfusion and capillary permeability. Phys Med Biol 2012; 57: R1–33. doi: 10.1088/0031-9155/57/2/R1 22173205

[18] Sourbron SP, Buckley DL . Classic models for dynamic contrast-enhanced MRI. NMR Biomed 2013; 26: 1004–27. doi: 10.1002/nbm.2940 23674304

[19] Ingrisch M, Sourbron S . Tracer-kinetic modeling of dynamic contrast-enhanced MRI and CT: a primer. J Pharmacokinet Pharmacodyn 2013; 40: 281–300. doi: 10.1007/s10928-013-9315-3 23563847

[20] Barnes SR, Ng TSC, Santa-Maria N, Montagne A, Zlokovic BV, Jacobs RE . ROCKETSHIP: a flexible and modular software tool for the planning, processing and analysis of dynamic MRI studies. BMC Med Imaging 2015; 15: 19. doi: 10.1186/s12880-015-0062-3 26076957 PMC4466867

[21] Berks M, Parker GJM, Little R, Cheung S . Madym: a C++ toolkit for quantitative DCE-MRI analysis. JOSS 2021; 6: 3523. doi: 10.21105/joss.03523

[22] Li X, Morgan PS, Ashburner J, Smith J, Rorden C . The first step for neuroimaging data analysis: DICOM to nifti conversion. J Neurosci Methods 2016; 264: 47–56. doi: 10.1016/j.jneumeth.2016.03.001 26945974

[23] Lin L-K . A concordance correlation coefficient to evaluate reproducibility. Biometrics 1989; 45: 255–68: 255. doi: 10.2307/2532051 2720055

[24] McBride G . A proposal for strength-of-agreement criteria for Lin’s concordance correlation coefficient. NIWA Client Report: HAM2005-062 2005; 45: 307–10.

[25] Beers A, Chang K, Brown J, Zhu X, Sengupta D, Willke TL, et al . Anatomical DCE-MRI phantoms generated from glioma patient data. In: Paper presented at the In: Paper presented at the In: Paper presented at the Physics of Medical Imaging, Houston, United States. doi: 10.1117/12.2294961

[26] Bernal J, Valdés-Hernández MDC, Escudero J, Heye AK, Sakka E, Armitage PA, et al . A four-dimensional computational model of dynamic contrast-enhanced magnetic resonance imaging measurement of subtle blood-brain barrier leakage. Neuroimage 2021; 230: 117786. doi: 10.1016/j.neuroimage.2021.117786 33497771 PMC8065875

[27] Semmineh NB, Stokes AM, Bell LC, Boxerman JL, Quarles CC . A population-based digital reference object (DRO) for optimizing dynamic susceptibility contrast (DSC) -MRI methods for clinical trials. Tomography 2017; 3: 41–49. doi: 10.18383/j.tom.2016.00286 28584878 PMC5454781

[28] Bliesener Y, Lingala SG, Haldar JP, Nayak KS . Impact of (K, T) sampling on DCE MRI tracer kinetic parameter estimation in digital reference objects. Magn Reson Med 2020; 83: 1625–39. doi: 10.1002/mrm.28024 31605556 PMC6982604

[29] Bosca RJ, Jackson EF . Creating an anthropomorphic digital Mr phantom -- an extensible tool for comparing and evaluating quantitative imaging algorithms. Phys Med Biol 2016; 61: 974–82. doi: 10.1088/0031-9155/61/2/974 26738776

[30] Debus C, Floca R, Ingrisch M, Kompan I, Maier-Hein K, Abdollahi A, et al . MITK-modelfit: a generic open-source framework for model fits and their exploration in medical imaging-design, implementation and application on the example of DCE-MRI. BMC Bioinformatics 2019; 20(): 31. doi: 10.1186/s12859-018-2588-1 30651067 PMC6335810

[31] Hansen MB, Tietze A, Haack S, Kallehauge J, Mikkelsen IK, Østergaard L, et al . Robust estimation of hemo-dynamic parameters in traditional DCE-MRI models. PLoS One 2019; 14(): e0209891. doi: 10.1371/journal.pone.0209891 30605459 PMC6317807

[32] Kim H, Mousa M, Schexnailder P, Hergenrother R, Bolding M, Ntsikoussalabongui B, et al . Portable perfusion phantom for quantitative DCE-MRI of the abdomen. Med Phys 2017; 44: 5198–5209. doi: 10.1002/mp.12466 28692137 PMC5646228

[33] Foltz W, Driscoll B, Laurence Lee S, Nayak K, Nallapareddy N, Fatemi A, et al . Phantom validation of DCE-MRI magnitude and phase-based vascular input function measurements. Tomography 2019; 5: 77–89. doi: 10.18383/j.tom.2019.00001 30854445 PMC6403037

[34] Cox RW, Ashburner J, Breman H, Fissell K, Haselgrove C, Holmes CJ . A (sort of) new image data format standard: NIfTI-1. 10th Annual Meeting of the Organization for Human Brain Mapping. Budapest: NeuroImage; 2004.

