# Supplementary material for: Open source code for the generation of digital reference objects for dynamic contrast-enhanced MRI analysis software validation
Source: Br J Radiol. 2023 May 25;96(1147):20220976. doi: 10.1259/bjr.20220976 (PMC10321261; doi:10.1259/bjr.20220976)
Supplement: Supplementary Material 1. [file bjr.20220976.suppl-01.docx]

**Open source code for the generation of digital reference objects for dynamic contrast enhanced MRI analysis software validation**

**Supplementary Material:-**

**Sample curve fitting plots for DRO voxels with non-perfect convergence**

Sample curve fits were plotted for cases of non-convergence or poor convergence of the model optimization process in each of the analysis software packages tested that provided results data with this information included and allowed setting of initial parameter estimates (i.e., MADYM and ROCKETSHIP). The MIStar software package did not permit manual setting of initial parameter estimates.

In all cases tested, poor convergence or non-convergence could be corrected by changing the initial parameter estimates fed into the optimizer. This indicated that the optimizer had found a local minimum in the original non-converging curve-fitting process.

Sample curve fits are shown in the figures below. In these figures, the results table columns shown display the observed (‘Obs.’), true (‘True’), relative percentage (‘Rel.’) and initial estimates (‘Init.’) of the parameters fitted by each model. In all cases, the time resolution of the dynamic series in the DRO was set to 0.5 s and the model algorithm integrations were performed also with a time resolution of 0.5 s.


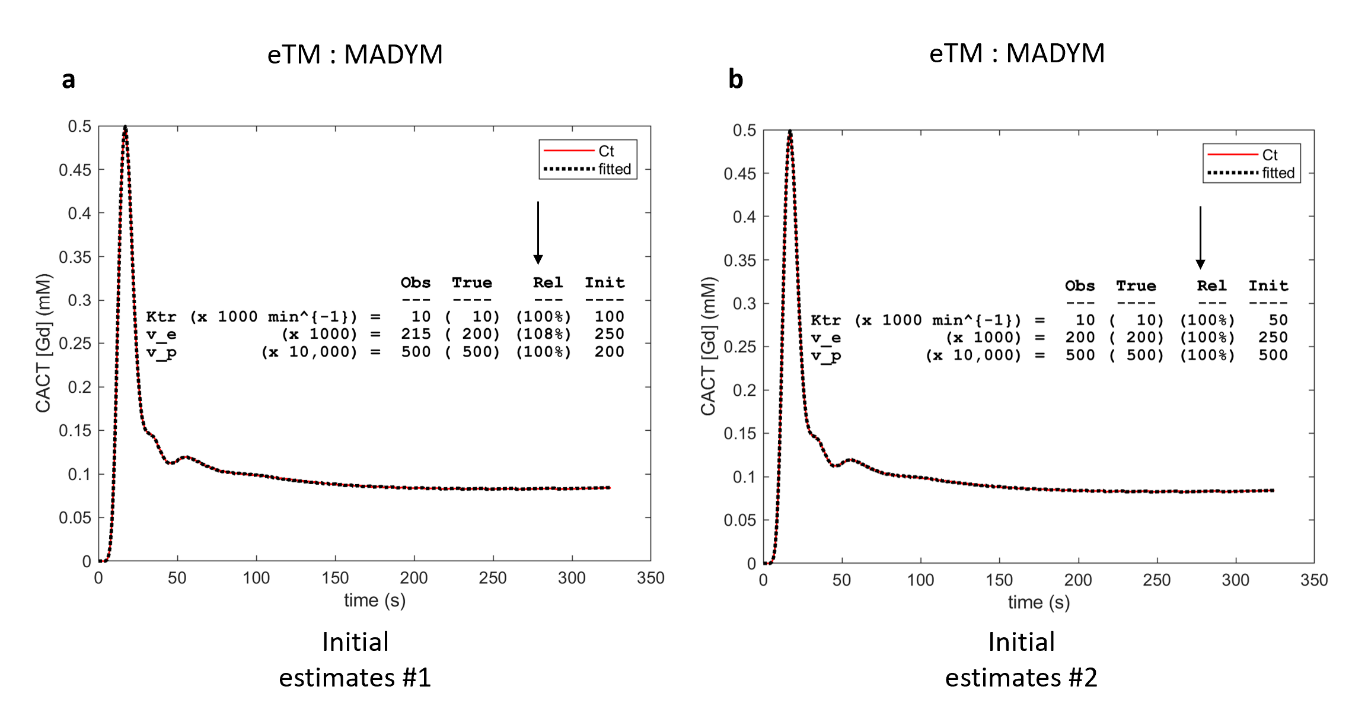


**Figure S1.** Sample curve fits from the eTM example DRO analyzed by MADYM, showing (a) a local minimum with initial parameter estimate set #1 and (b) a good fit in the same voxel with a different set of initial parameter estimates (#2). This was one of the very few voxel curve fits in the eTM DRO that failed to converge perfectly with initial parameter estimates set #1.


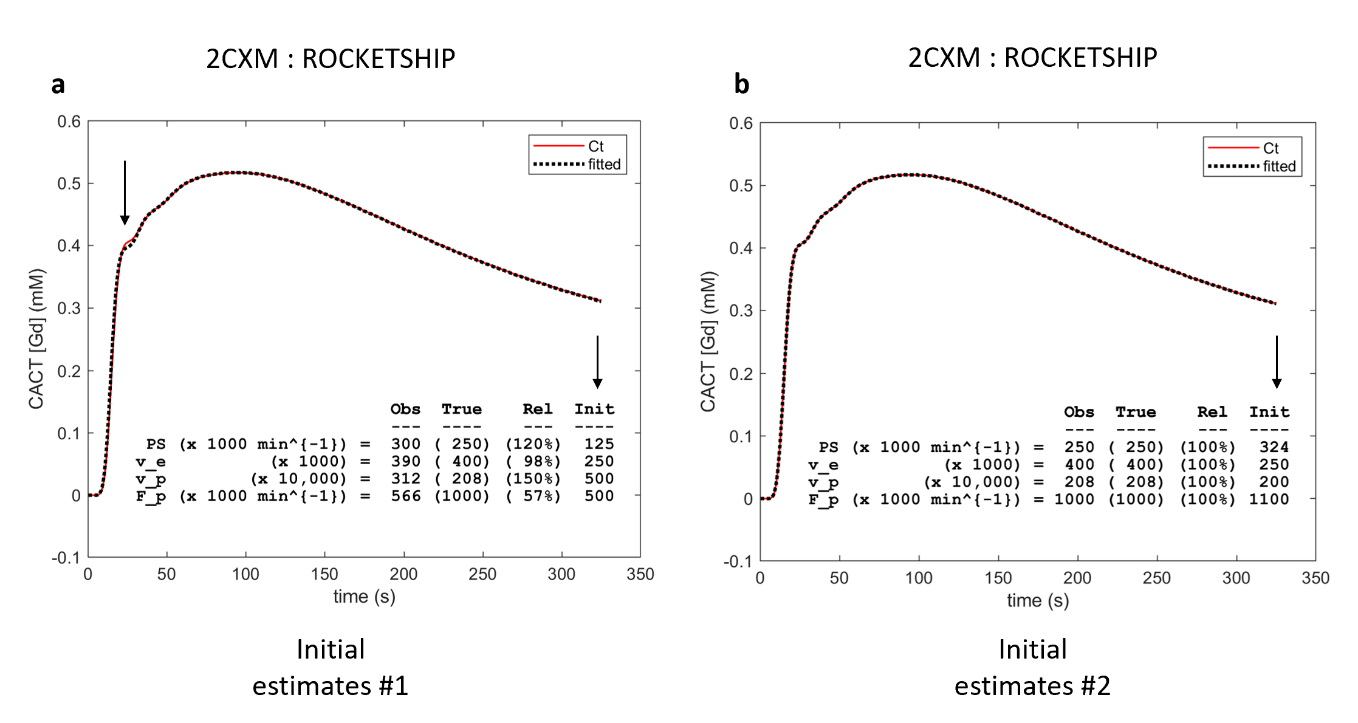


**Figure S2.** Sample curve fits from the 2CXM example DRO analyzed by ROCKETSHIP, showing (a) a sub-optimal fitted curve with a local minimum in fitted parameters. This is corrected in the same voxel (b) when different initial parameter estimates were used in the model-fitting process.


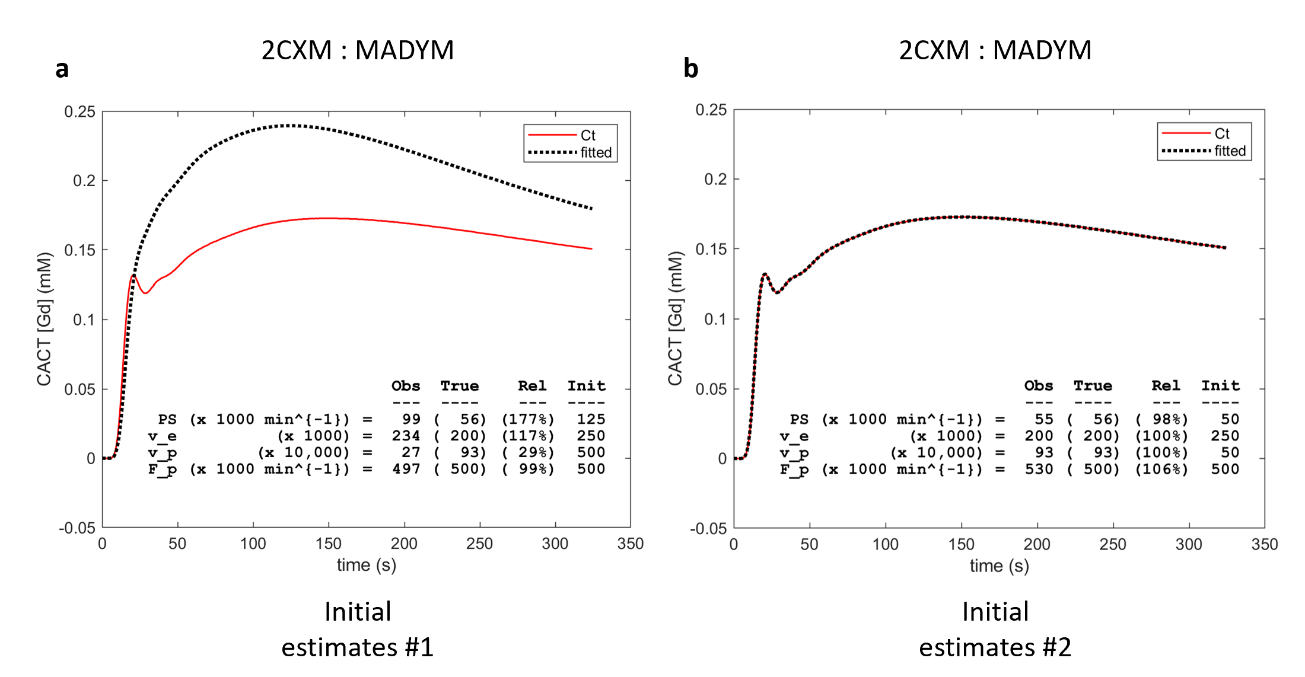


**Figure S3.** Sample curve fits from a voxel in the 2CXM example DRO analyzed by MADYM, showing (a) ‘worst-case’ curve-fitting. This is substantially, though not perfectly, corrected by a change in initial parameter estimates (b).


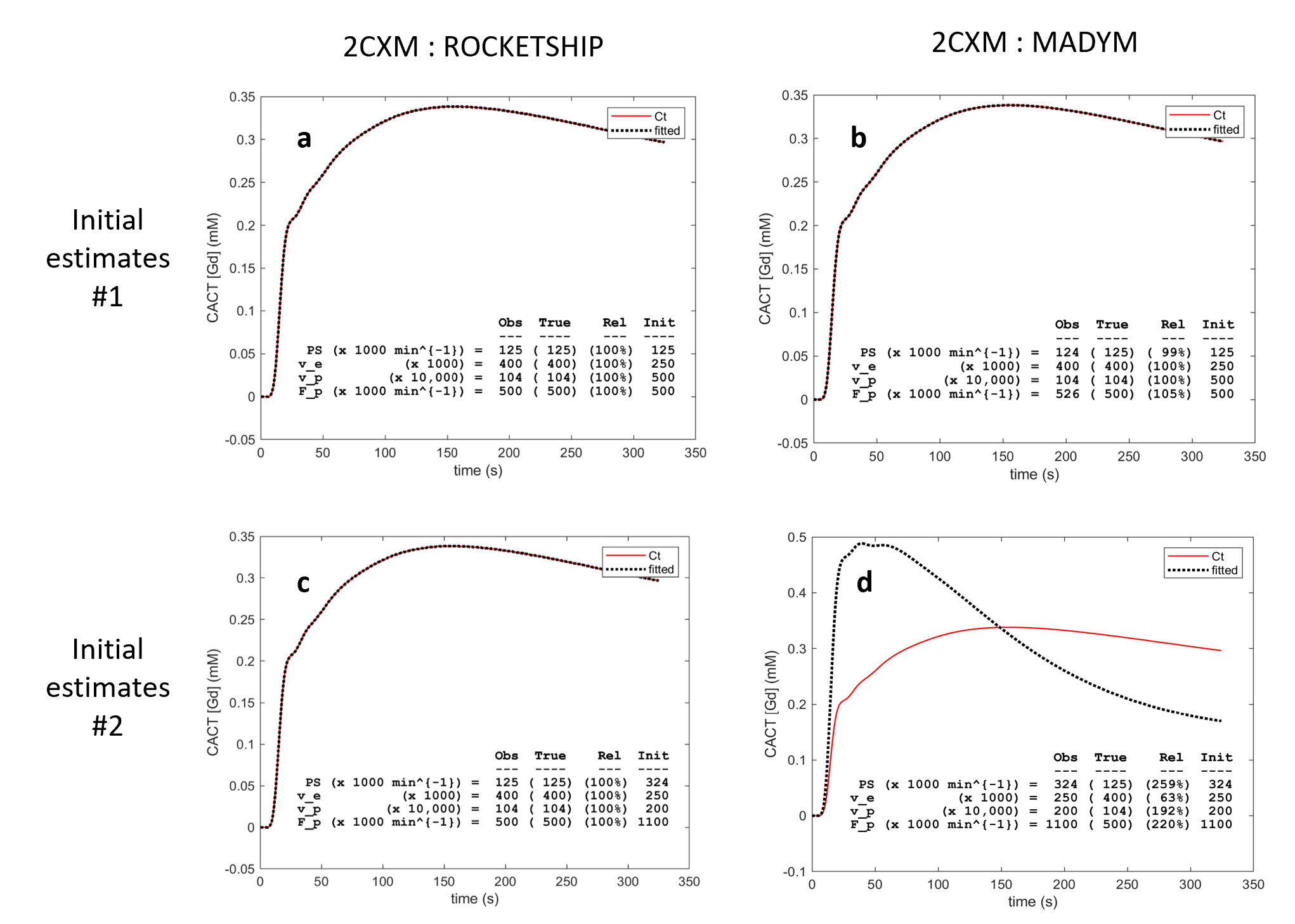


**Figure S4.** Sample curve fits from the same voxel in the 2CXM DRO when analyzed using ROCKETSHIP and MADYM with two different sets of initial parameter estimates. ROCKETSHIP shows (a & c) perfect convergence for both sets of initial parameter estimates; MADYM shows convergence (b) with ‘good’ initial parameter estimates and lack of convergence (d) when the initial parameter estimates are very different from the true values. This indicates that ROCKETSHIP was more robust in convergence than MADYM when analyzing this 2CXM DRO. The reasons for this are unclear but may reflect the fact that the DRO was generated using a similarly coded algorithm for the model as that employed by ROCKETSHIP. Non-convergence in curve fitting was the exception rather than the rule in the 2CXM DRO for both software packages tested, as indicated by Figure 3 in the main manuscript.
